# (*E*)-4-(5-Hydr­oxy-2-methyl­benzyl­idene­amino)-1,5-dimethyl-2-phenyl-1*H*-pyrazol-3(2*H*)-one

**DOI:** 10.1107/S1600536808029930

**Published:** 2008-09-27

**Authors:** Yun-Fa Zheng, Ming-Hua Yang

**Affiliations:** a Department of Chemistry, Lishui University, Lishui 323000, People’s Republic of China

## Abstract

The title compound, C_19_H_19_N_3_O_2_, is a Schiff base compound derived from 4-amino­anti­pyrine and 5-hydr­oxy-2-methyl­benzaldehyde. The mol­ecule adopts a *trans* configuration about the central C=N bond. There is an intra­molecular O—H⋯N hydrogen bond. Futhermore, weak C—H⋯O hydrogen bonds lead to the formation of a chain developing parallel to the *b* axis.

## Related literature

For related literature, see: Alemi & Shaabani (2000 ▶); Kim & Shin (1999 ▶); Yan *et al.* (2006 ▶); Zheng *et al.* (2006 ▶); You *et al.* (2006 ▶).
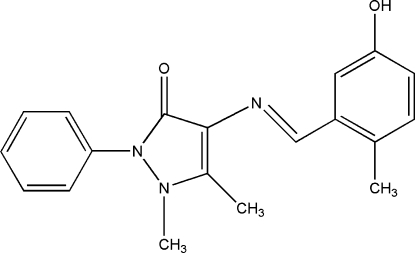


## Experimental

### 

#### Crystal data

C_19_H_19_N_3_O_2_
*M* *_r_* = 321.37
Monoclinic, 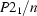

*a* = 12.030 (2) Å
*b* = 7.1400 (14) Å
*c* = 20.210 (4) Å
β = 104.01 (3)°
*V* = 1684.4 (6) Å^3^
*Z* = 4
Mo *K*α radiation
μ = 0.08 mm^−1^
*T* = 298 (2) K
0.28 × 0.27 × 0.23 mm

#### Data collection

Bruker APEXII area-detector diffractometer
Absorption correction: multi-scan (*SADABS*; Sheldrick, 1996 ▶) *T*_min_ = 0.965, *T*_max_ = 0.971
12992 measured reflections
3038 independent reflections
2101 reflections with *I* > 2σ(*I*)
*R*_int_ = 0.025

#### Refinement

*R*[*F* ^2^ > 2σ(*F* ^2^)] = 0.037
*wR*(*F* ^2^) = 0.117
*S* = 1.11
3038 reflections
220 parameters
H-atom parameters constrained
Δρ_max_ = 0.19 e Å^−3^
Δρ_min_ = −0.18 e Å^−3^

### 

Data collection: *SMART* (Bruker, 1998 ▶); cell refinement: *SAINT* (Bruker, 1999 ▶); data reduction: *SAINT*; program(s) used to solve structure: *SHELXS97* (Sheldrick, 2008 ▶); program(s) used to refine structure: *SHELXL97* (Sheldrick, 2008 ▶); molecular graphics: *ORTEPIII* (Burnett & Johnson, 1996 ▶), *ORTEP-3 for Windows* (Farrugia, 1997 ▶) and *PLATON* (Spek, 2003 ▶); software used to prepare material for publication: *SHELXL97* (Sheldrick, 2008 ▶).

## Supplementary Material

Crystal structure: contains datablocks I, global. DOI: 10.1107/S1600536808029930/dn2375sup1.cif

Structure factors: contains datablocks I. DOI: 10.1107/S1600536808029930/dn2375Isup2.hkl

Additional supplementary materials:  crystallographic information; 3D view; checkCIF report

## Figures and Tables

**Table 1:** Hydrogen-bond geometry (Å, °)

*D*—H⋯*A*	*D*—H	H⋯*A*	*D*⋯*A*	*D*—H⋯*A*
O2—H2⋯N1	0.82	1.90	2.6275 (19)	148
C10—H10*C*⋯O1^i^	0.96	2.46	3.386 (2)	163

Symmetry code: (i) 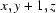.

**Fig. 1.**
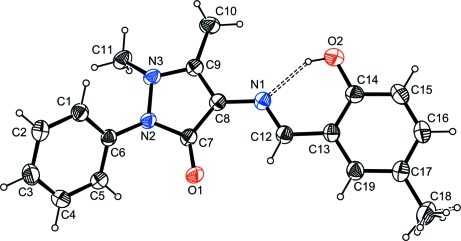
Molecular structure of (I), showing the atom-labelling scheme. Ellipsoids are drawn at the 30% probability level. H atoms are represented as small spheres of arbitrary radii. Hydrogen bond is shown as dashed line.

**Fig. 2.**
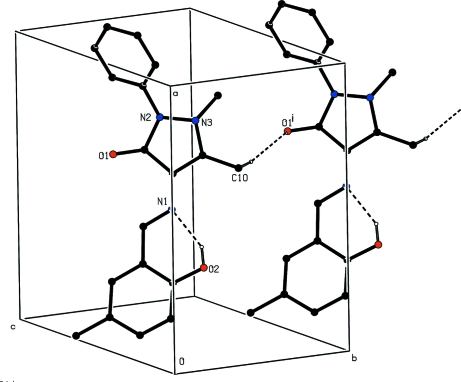
Partial packing view showing the formation of the chain through C-H···O hydrogen bondings displayed as dashed line. H atoms not involved in hydrogen bonds have been omitted for clarity. [Symmetry code: (i) x, 1+y, z]

## supplementary crystallographic information

### Comment

The design, synthesis, characterization, and properties of Schiff bases and Schiff base complexes. (Yan *et al.*, 2006; Zheng *et al.*, 2006; You *et al.*, 2006) are still of great interest. Schiff bases that have solvent dependent UV/vis spectra (solvatochromicity) can be suitable NLO active materials (Alemi & Shaabani, 2000). They are also useful in asymmetric oxidation of methyl phenyl sulfide (Kim & Shin, 1999).

The molecule adopts trans configuration about the central C=N bond (Fig. 1). There is an intramolecular O-H···N hydrogen bond. Futhermore, weak C-H···O hydrogen bonds lead to the formation of a chain developping parallel to the b axis (Table 1, Fig. 2).

### Experimental

Under nitrogen, a mixture of 5-hydroxy-2-methylbenzaldehyde (1.36 g,10 mmol) and 4-amino-1,2-dihydro-1,5-dimethyl -1-phenylpyrazol-3-one (2.03 g, 10 mmol) in absolute ethanol (120 ml) was refluxed for about 3 h to yield a yellow precipitate. The product was collected by vacuum filtration and washed with ethanol. The crude solid was redissolved in CH_2_Cl_2_ (100 ml) and washed with water (2*10 ml)and brine(10 ml). After dried over Na_2_SO_4_, the solvent was removed under vacuum, and yellow solid was isolated in yield 92% (3.5 g). Colourless single crystals of the compound suitable for X-ray analysis were grown from CH_2_Cl_2_ and absolute ethanol(5:1) by slow evaporation of the solvent at room temperature over a period of about a week.

### Refinement

All H atoms attached to C and O atoms were fixed geometrically and treated as riding with C—H = 0.96 Å (methyl) or 0.93 Å (aromatic) and O—H = 0.82 Å with U_iso_(H) = 1.2U_eq_(aromatic) or U_iso_(H) = 1.5U_eq_(methyl, O). The H attached to C18 are statistically disordered over two positions.

### Crystal data

**Table:**

C_19_H_19_N_3_O_2_	*F*(000) = 680
*M**_r_* = 321.37	*D*_x_ = 1.267 Mg m^−^^3^
Monoclinic, *P*2_1_/*n*	Mo *K*α radiation, λ = 0.71073 Å
Hall symbol: -P 2yn	Cell parameters from 3038 reflections
*a* = 12.030 (2) Å	θ = 3.0–25.2°
*b* = 7.1400 (14) Å	µ = 0.08 mm^−^^1^
*c* = 20.210 (4) Å	*T* = 298 K
β = 104.01 (3)°	Block, colourless
*V* = 1684.4 (6) Å^3^	0.28 × 0.27 × 0.23 mm
*Z* = 4	

### Data collection

**Table:**

Bruker APEXII area-detector diffractometer	3038 independent reflections
Radiation source: fine-focus sealed tube	2101 reflections with *I* > 2σ(*I*)
graphite	*R*_int_ = 0.026
φ and ω scans	θ_max_ = 25.2°, θ_min_ = 3.0°
Absorption correction: multi-scan (SADABS; Sheldrick, 1996)	*h* = −14→14
*T*_min_ = 0.965, *T*_max_ = 0.971	*k* = −8→8
12992 measured reflections	*l* = −24→24

### Refinement

**Table:**

Refinement on *F*^2^	Primary atom site location: structure-invariant direct methods
Least-squares matrix: full	Secondary atom site location: difference Fourier map
*R*[*F*^2^ > 2σ(*F*^2^)] = 0.037	Hydrogen site location: inferred from neighbouring sites
*wR*(*F*^2^) = 0.117	H-atom parameters constrained
*S* = 1.11	*w* = 1/[σ^2^(*F*_o_^2^) + (0.0593*P*)^2^ + 0.1685*P*] where *P* = (*F*_o_^2^ + 2*F*_c_^2^)/3
3038 reflections	(Δ/σ)_max_ = 0.003
220 parameters	Δρ_max_ = 0.19 e Å^−^^3^
0 restraints	Δρ_min_ = −0.18 e Å^−^^3^

### Special details

**Table:**

Geometry. All esds (except the esd in the dihedral angle between two l.s. planes) are estimated using the full covariance matrix. The cell esds are taken into account individually in the estimation of esds in distances, angles and torsion angles; correlations between esds in cell parameters are only used when they are defined by crystal symmetry. An approximate (isotropic) treatment of cell esds is used for estimating esds involving l.s. planes.
Refinement. Refinement of *F*^2^ against ALL reflections. The weighted *R*-factor *wR* and goodness of fit *S* are based on *F*^2^, conventional *R*-factors *R* are based on *F*, with *F* set to zero for negative *F*^2^. The threshold expression of *F*^2^ > σ(*F*^2^) is used only for calculating *R*-factors(gt) *etc*. and is not relevant to the choice of reflections for refinement. *R*-factors based on *F*^2^ are statistically about twice as large as those based on *F*, and *R*- factors based on ALL data will be even larger.

### Fractional atomic coordinates and isotropic or equivalent isotropic displacement parameters (Å^2^)

**Table:**

	*x*	*y*	*z*	*U*_iso_*/*U*_eq_	Occ. (<1)
N1	0.54536 (10)	0.08488 (19)	0.09989 (7)	0.0484 (4)	
N2	0.84821 (10)	0.10229 (18)	0.18740 (7)	0.0478 (4)	
N3	0.82904 (11)	0.27459 (18)	0.15274 (7)	0.0489 (4)	
O1	0.74035 (9)	−0.16626 (16)	0.18989 (7)	0.0607 (4)	
O2	0.35066 (11)	0.19101 (19)	0.01845 (7)	0.0721 (4)	
H2	0.4177	0.1999	0.0399	0.108*	
C1	1.03702 (13)	0.0611 (2)	0.16517 (8)	0.0500 (4)	
H1	1.0178	0.1448	0.1289	0.060*	
C2	1.14339 (14)	−0.0241 (3)	0.18084 (9)	0.0569 (5)	
H2A	1.1959	0.0045	0.1553	0.068*	
C3	1.17288 (15)	−0.1505 (3)	0.23369 (11)	0.0659 (5)	
H3	1.2445	−0.2076	0.2436	0.079*	
C4	1.09553 (15)	−0.1913 (3)	0.27139 (10)	0.0667 (5)	
H4	1.1144	−0.2776	0.3068	0.080*	
C5	0.98929 (14)	−0.1047 (2)	0.25717 (9)	0.0549 (5)	
H5	0.9378	−0.1311	0.2836	0.066*	
C6	0.95980 (12)	0.0212 (2)	0.20351 (8)	0.0433 (4)	
C7	0.74648 (13)	−0.0029 (2)	0.17136 (9)	0.0469 (4)	
C8	0.66180 (13)	0.1204 (2)	0.13035 (8)	0.0454 (4)	
C9	0.71364 (13)	0.2853 (2)	0.12208 (8)	0.0472 (4)	
C10	0.66222 (17)	0.4584 (2)	0.08666 (11)	0.0650 (5)	
H10A	0.5826	0.4372	0.0659	0.097*	
H10B	0.7013	0.4912	0.0521	0.097*	
H10C	0.6696	0.5588	0.1191	0.097*	
C11	0.89730 (16)	0.4347 (2)	0.18612 (10)	0.0618 (5)	
H11A	0.8869	0.5390	0.1552	0.093*	
H11B	0.9768	0.4006	0.1986	0.093*	
H11C	0.8727	0.4689	0.2263	0.093*	
C12	0.49748 (13)	−0.0712 (2)	0.10893 (9)	0.0494 (4)	
H12	0.5403	−0.1634	0.1362	0.059*	
C13	0.37643 (13)	−0.1053 (2)	0.07677 (8)	0.0469 (4)	
C14	0.30773 (14)	0.0258 (2)	0.03279 (9)	0.0524 (4)	
C15	0.19260 (15)	−0.0150 (3)	0.00338 (9)	0.0630 (5)	
H15	0.1475	0.0710	−0.0258	0.076*	
C16	0.14615 (15)	−0.1806 (3)	0.01736 (9)	0.0625 (5)	
H16	0.0694	−0.2050	−0.0025	0.075*	
C17	0.21076 (14)	−0.3139 (3)	0.06057 (9)	0.0581 (5)	
C18	0.15794 (18)	−0.4936 (3)	0.07672 (13)	0.0859 (7)	
H18A	0.2150	−0.5676	0.1070	0.129*	0.50
H18B	0.1283	−0.5621	0.0353	0.129*	0.50
H18C	0.0968	−0.4660	0.0981	0.129*	0.50
H18D	0.0784	−0.4962	0.0533	0.129*	0.50
H18E	0.1651	−0.5017	0.1250	0.129*	0.50
H18F	0.1966	−0.5978	0.0622	0.129*	0.50
C19	0.32577 (13)	−0.2731 (2)	0.08928 (9)	0.0546 (4)	
H19	0.3703	−0.3611	0.1178	0.066*	

### Atomic displacement parameters (Å^2^)

**Table:**

	*U*^11^	*U*^22^	*U*^33^	*U*^12^	*U*^13^	*U*^23^
N1	0.0449 (8)	0.0456 (8)	0.0570 (8)	0.0014 (6)	0.0167 (6)	−0.0045 (6)
N2	0.0432 (7)	0.0360 (7)	0.0657 (9)	−0.0001 (6)	0.0162 (6)	0.0038 (6)
N3	0.0522 (8)	0.0338 (7)	0.0632 (9)	−0.0023 (6)	0.0190 (7)	0.0010 (6)
O1	0.0527 (7)	0.0390 (7)	0.0894 (9)	−0.0031 (5)	0.0155 (6)	0.0106 (6)
O2	0.0633 (8)	0.0671 (9)	0.0836 (10)	0.0044 (7)	0.0131 (7)	0.0225 (7)
C1	0.0485 (9)	0.0541 (10)	0.0469 (9)	−0.0024 (8)	0.0108 (7)	−0.0004 (8)
C2	0.0462 (9)	0.0640 (12)	0.0617 (11)	−0.0030 (8)	0.0157 (8)	−0.0062 (9)
C3	0.0460 (10)	0.0657 (13)	0.0818 (14)	0.0045 (9)	0.0072 (9)	0.0020 (11)
C4	0.0594 (11)	0.0615 (12)	0.0729 (13)	0.0021 (9)	0.0040 (9)	0.0177 (10)
C5	0.0528 (10)	0.0541 (11)	0.0582 (11)	−0.0057 (8)	0.0139 (8)	0.0056 (9)
C6	0.0412 (8)	0.0386 (9)	0.0492 (9)	−0.0047 (7)	0.0092 (7)	−0.0046 (7)
C7	0.0444 (9)	0.0396 (9)	0.0596 (10)	−0.0003 (7)	0.0180 (7)	−0.0028 (8)
C8	0.0449 (9)	0.0402 (9)	0.0543 (10)	0.0023 (7)	0.0182 (7)	−0.0031 (7)
C9	0.0494 (9)	0.0414 (9)	0.0536 (10)	0.0036 (7)	0.0176 (7)	−0.0031 (7)
C10	0.0704 (12)	0.0459 (11)	0.0794 (13)	0.0078 (9)	0.0196 (10)	0.0107 (9)
C11	0.0649 (11)	0.0410 (10)	0.0824 (13)	−0.0108 (8)	0.0234 (10)	−0.0065 (9)
C12	0.0448 (9)	0.0455 (10)	0.0584 (10)	0.0048 (7)	0.0137 (7)	−0.0030 (8)
C13	0.0424 (8)	0.0487 (10)	0.0507 (9)	0.0034 (7)	0.0135 (7)	−0.0051 (8)
C14	0.0536 (10)	0.0530 (11)	0.0520 (10)	0.0042 (8)	0.0152 (8)	0.0028 (8)
C15	0.0528 (10)	0.0784 (14)	0.0532 (11)	0.0096 (10)	0.0042 (8)	0.0047 (10)
C16	0.0475 (10)	0.0798 (14)	0.0563 (11)	−0.0026 (9)	0.0051 (8)	−0.0087 (10)
C17	0.0498 (9)	0.0611 (11)	0.0628 (11)	−0.0055 (9)	0.0122 (8)	−0.0103 (9)
C18	0.0655 (13)	0.0754 (15)	0.1124 (18)	−0.0206 (11)	0.0128 (12)	−0.0031 (13)
C19	0.0469 (9)	0.0509 (10)	0.0647 (11)	0.0027 (8)	0.0108 (8)	−0.0023 (9)

### Geometric parameters (Å, °)

**Table:**

N1—C12	1.288 (2)	C10—H10A	0.9600
N1—C8	1.410 (2)	C10—H10B	0.9600
N2—C7	1.405 (2)	C10—H10C	0.9600
N2—N3	1.4068 (18)	C11—H11A	0.9600
N2—C6	1.425 (2)	C11—H11B	0.9600
N3—C9	1.379 (2)	C11—H11C	0.9600
N3—C11	1.472 (2)	C12—C13	1.464 (2)
O1—C7	1.2330 (19)	C12—H12	0.9300
O2—C14	1.347 (2)	C13—C19	1.395 (2)
O2—H2	0.8200	C13—C14	1.412 (2)
C1—C6	1.376 (2)	C14—C15	1.399 (2)
C1—C2	1.383 (2)	C15—C16	1.366 (3)
C1—H1	0.9300	C15—H15	0.9300
C2—C3	1.378 (3)	C16—C17	1.394 (3)
C2—H2A	0.9300	C16—H16	0.9300
C3—C4	1.369 (3)	C17—C19	1.395 (2)
C3—H3	0.9300	C17—C18	1.503 (3)
C4—C5	1.386 (3)	C18—H18A	0.9600
C4—H4	0.9300	C18—H18B	0.9600
C5—C6	1.387 (2)	C18—H18C	0.9600
C5—H5	0.9300	C18—H18D	0.9600
C7—C8	1.446 (2)	C18—H18E	0.9600
C8—C9	1.361 (2)	C18—H18F	0.9600
C9—C10	1.486 (2)	C19—H19	0.9300
			
C12—N1—C8	121.67 (14)	H11A—C11—H11C	109.5
C7—N2—N3	108.89 (12)	H11B—C11—H11C	109.5
C7—N2—C6	123.74 (13)	N1—C12—C13	120.77 (15)
N3—N2—C6	120.05 (12)	N1—C12—H12	119.6
C9—N3—N2	107.34 (12)	C13—C12—H12	119.6
C9—N3—C11	123.52 (14)	C19—C13—C14	117.94 (15)
N2—N3—C11	116.40 (13)	C19—C13—C12	119.56 (15)
C14—O2—H2	109.5	C14—C13—C12	122.50 (16)
C6—C1—C2	119.58 (16)	O2—C14—C15	118.85 (16)
C6—C1—H1	120.2	O2—C14—C13	121.29 (15)
C2—C1—H1	120.2	C15—C14—C13	119.86 (17)
C3—C2—C1	121.07 (18)	C16—C15—C14	120.31 (17)
C3—C2—H2A	119.5	C16—C15—H15	119.8
C1—C2—H2A	119.5	C14—C15—H15	119.8
C4—C3—C2	119.25 (17)	C15—C16—C17	121.76 (17)
C4—C3—H3	120.4	C15—C16—H16	119.1
C2—C3—H3	120.4	C17—C16—H16	119.1
C3—C4—C5	120.45 (18)	C16—C17—C19	117.67 (17)
C3—C4—H4	119.8	C16—C17—C18	121.21 (17)
C5—C4—H4	119.8	C19—C17—C18	121.12 (18)
C4—C5—C6	119.99 (17)	C17—C18—H18A	109.5
C4—C5—H5	120.0	C17—C18—H18B	109.5
C6—C5—H5	120.0	H18A—C18—H18B	109.5
C1—C6—C5	119.64 (15)	C17—C18—H18C	109.5
C1—C6—N2	120.95 (15)	H18A—C18—H18C	109.5
C5—C6—N2	119.37 (14)	H18B—C18—H18C	109.5
O1—C7—N2	123.25 (15)	C17—C18—H18D	109.5
O1—C7—C8	131.64 (15)	H18A—C18—H18D	141.1
N2—C7—C8	105.10 (14)	H18B—C18—H18D	56.3
C9—C8—N1	122.57 (15)	H18C—C18—H18D	56.3
C9—C8—C7	108.46 (14)	C17—C18—H18E	109.5
N1—C8—C7	128.93 (15)	H18A—C18—H18E	56.3
C8—C9—N3	109.78 (14)	H18B—C18—H18E	141.1
C8—C9—C10	128.96 (15)	H18C—C18—H18E	56.3
N3—C9—C10	121.26 (15)	H18D—C18—H18E	109.5
C9—C10—H10A	109.5	C17—C18—H18F	109.5
C9—C10—H10B	109.5	H18A—C18—H18F	56.3
H10A—C10—H10B	109.5	H18B—C18—H18F	56.3
C9—C10—H10C	109.5	H18C—C18—H18F	141.1
H10A—C10—H10C	109.5	H18D—C18—H18F	109.5
H10B—C10—H10C	109.5	H18E—C18—H18F	109.5
N3—C11—H11A	109.5	C13—C19—C17	122.45 (16)
N3—C11—H11B	109.5	C13—C19—H19	118.8
H11A—C11—H11B	109.5	C17—C19—H19	118.8
N3—C11—H11C	109.5		

### Hydrogen-bond geometry (Å, °)

**Table:**

*D*—H···*A*	*D*—H	H···*A*	*D*···*A*	*D*—H···*A*
O2—H2···N1	0.82	1.90	2.6275 (19)	148.
C10—H10C···O1^i^	0.96	2.46	3.386 (2)	163.

Symmetry codes: (i) *x*, *y*+1, *z*.
